# Nile Tilapia Derived TP4 Shows Broad Cytotoxicity toward to Non-Small-Cell Lung Cancer Cells

**DOI:** 10.3390/md16120506

**Published:** 2018-12-13

**Authors:** Chen-Hung Ting, Jyh-Yih Chen

**Affiliations:** 1 Marine Research Station, Institute of Cellular and Organismic Biology, Academia Sinica, Ilan 262, Taiwan; koichiting@gmail.com; 2 The iEGG and Animal Biotechnology Center, National Chung Hsing University, Taichung 402, Taiwan

**Keywords:** Antimicrobial peptide (AMP), Tilapia piscidin 4 (TP4), non-small cell lung cancer (NSCLC)

## Abstract

Non-small cell lung cancer (NSCLC) is among the leading causes of human mortality due to a lack of effective treatments. Conventional chemotherapies affect healthy cells and cause multidrug resistance, while tumors may eventually develop resistance to less-toxic targeted therapies. Thus, the need to develop novel therapies for NSCLC is urgent. Here, we show that Nile tilapia-derived Tilapia piscidin (TP) 4 is cytotoxic to a panel of NSCLC cells with different genetic profiles. We observed that TP4 triggers NSCLC cell death through the necrosis and combining TP4 with potent Epidermal growth factor receptor (EGFR)- tyrosine kinase inhibitors (TKI)s, Erlotinib, and Gefitinib, improved drug responses in EGFR-mutated NSCLC cells, but not in EGFR-wild-type NSCLC cells. This work provides novel insights into potential NSCLC treatments, which may utilize antimicrobial peptide TP4 as monotherapy or in combination with EGFR-TKIs.

## 1. Introduction

Lung cancer is the leading cause of cancer mortality worldwide [1]. Most lung cancer patients die within one year of diagnosis, and the five-year-survival rate is around 18.6% [2]. Among these patients, over 85% are diagnosed with non-small cell lung cancer (NSCLC), while 15% have small cell lung cancer (SCLC). About 50% of NSCLCs are phenotypically characterized as adenocarcinomas (ADCs), of which gene expression profiles are consistent with a distal lung cell origin. Meanwhile, about 40% of NSCLCs are squamous cell carcinomas (SCCs), which are thought to arise from proximal tracheal basal cells in the lung [3,4]. Chemotherapies are routinely used for NSCLC treatment; however, chemotherapeutic drugs can cause serious side-effects and multidrug resistance. Advanced transcriptome analyses unveiled several gene mutations that are associated with NSCLC, including Epidermal growth factor receptor (EGFR) [5,6,7,8]. Constitutively activating EGFR mutations have been observed in 10% and 35% of NSCLC patients in the U.S. and East Asia, respectively [9,10]. Due to the tumorigenic nature of these mutations, EGFR-directed tyrosine kinase inhibitors (TKIs) have become valuable tools for the treatment of NSCLC [6,7,8,11,12,13,14]. The first and second-generation EGFR-TKIs (e.g., Erlotinib, Gefitinib, and Afatinib) were developed to target proteins derived from exon 19 deletions or a point mutation in exon 21 (L858R) [9]. These early EGFR-TKIs have produced remarkable results in the clinic; however, the median progression-free survival (PFS) is still less than 12 months due to the occurrence of drug-resistant cancer cells [15,16]. One critical secondary mutation that confers drug resistance is a gatekeeper point mutation in exon 20 (T790M) of EGFR, which is observed in about 50%–60% of all patients [11,17,18,19]. The third-generation EGFR-TKIs (e.g. Osimertinib) were developed to selectively target mutants (particularly the T790M mutation) and show low affinity to wild-type EGFR, thereby significantly reducing toxicity [13,20]. Unfortunately, other novel mutations may still occur in TKI-treated tumors, leading to drug resistance [21,22]. In order to overcome this resistance, combinations of EGFR-TKIs with chemotherapy or other targeted agents are recommended [20,23,24]. Thus, alternative strategies and novel drugs for treatment of NSCLC are urgently needed.

Antimicrobial peptides (AMPs) are evolutionarily conserved peptides that function to combat microbial infections [25] and have been suggested as potential anti-cancer agents [26,27,28]. The defensive utility of cationic AMPs is derived from a structural amphipathic property, which enables electrostatic interactions with anionic molecules on the plasma membrane of microbes or cancer cells [26,27,28]. Unlike chemotherapeutic drugs, which damage healthy cells, AMPs selectively target cancer cells with lower toxicity to non-cancerous cell types [26,27,28]. We previously found that the Nile tilapia (*Oreochromis niloticus*)-derived cationic AMP, tilapia piscidin (TP4) [29], binds to the negatively charged membrane of breast cancer cells and subsequently triggers cancer cell death [27]. NSCLC cells are also good candidate targets for TP4 because extensive glycosylation and low cholesterol levels enhance the negative charge of the membrane [30,31].

In this study, we aimed to investigate the therapeutic potential of TP4 in NSCLC. We show that TP4 is highly cytotoxic to multiple NSCLC cell lines with different EGFR status. Moreover, NSCLC cells with wild-type EGFR were equally susceptible to TP4 alone and in combination with EGFR-TKIs, but combined treatment of TP4 with EGFR-TKIs showed enhanced cytotoxicity over TKIs or TP4 alone in EGFR-mutated NSCLC cells. Furthermore, TP4 was found to induce necrotic death in NSCLC cells. Combining TP4 with EGFR-TKIs enhanced necrosis in NSCLC cells with EGFR mutations. These findings support the notion that TP4 is a promising candidate drug for treatment of various NSCLC types.

## 2. Results

### 2.1. Cationic TP4 is Toxic to NSCLC Cells

**Figure 1:**
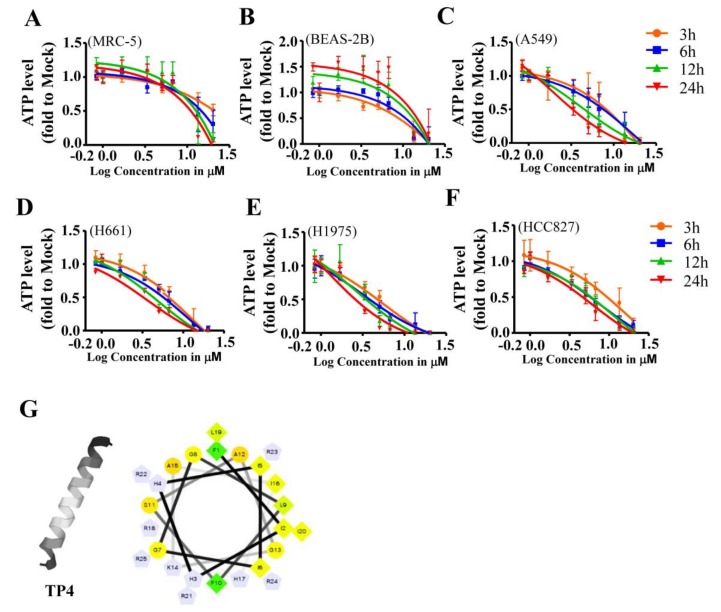
Broad cytotoxicity of Tilapia piscidin (TP) 4 toward non-small cell lung cancer (NSCLC) cells. (**A**–**F**) Cell viability in BEAS-2B (**A**), MRC-5 (**B**), and NSCLC cells (**C**–**F**) was determined by an adenosine triphosphate (ATP) assay, following treatment with varying doses of TP4 (0.83–20.12 μM) at the indicated time-points (3–24 h). The x-axis shows the logarithm of the TP4 concentration. Multiple wells were analyzed for each assay and compiled with other independent assays. Quantitative results represent the mean ± SD (statistical analyses are shown in Supplementary Tables S1 and S2). (**G**) The α-helical (left) and three-dimensional structures (right) of TP4. Relative position of each amino acid residues along the helix are numbered and connected with lines. Diamonds are presented as the hydrophobic residues; circles are hydrophilic residues. Triangles and pentagons are presented as residues with negatively charged and positively charged, respectively. Hydrophobicity is color-coded. The most hydrophobic residue is shown in green and the most hydrophilic residue is shown in red. Amino acid with zero hydrophobicity is shown in yellow.

Cancerous cells with negatively charged membranes might be attacked by AMPs [26,27,28]. To investigate whether NSCLC cells are potential targets for TP4, cytotoxicity was evaluated in a panel of NSCLC cell lines. Control cell lines, BEAS-2B and MRC-5, treated with a range of TP4 concentrations (0.838 to 6.710 μM) showed limited cytotoxicity, with 50% inhibitory concentrations (IC_50_) of over 15.48 and 26.50 μM, respectively (Figure 1A,B, Table 1). Notably, low TP4 concentrations, from 0.830 to 6.710 μM, enhanced cell proliferation in normal BEAS-2B cells, as evidenced by the gradual increase in relative adenosine triphoshapte (ATP) level of TP4-treated cells compared to mock-treated controls at 12 and 24 h post-TP4 treatment (Figure 1B). The IC_50_ values among NSCLC cells at different time points after TP4 treatment were 1.922–27.62 μM in A549 cells, 3.769–14.17 μM in NCI-H661 cells, 1.241–5.472 μM in NCI-H1975 cells, and 10.61–18.52 μM in HCC827 cells (Figure 1C–F, and Table 2).

### 2.2. Combining TP4 with Potent EGFR Tyrosine Kinase Inhibitors (TKIs) Enhances Toxicity

**Figure 2:**
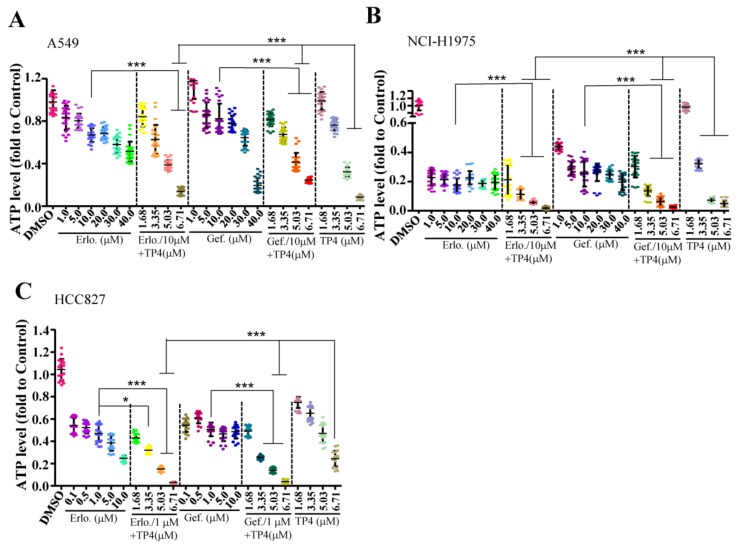
Combining TP4 and EGFR-TKIs enhances cytotoxicity. (**A**–**C**) Cell viability of A549 (**A**), H1975 cells (**B**), and HCC827 (**C**) were determined by the ATP assay 24 h after treatment with varying doses of EGFR-TKIs, TP4, or combinations thereof. At least six wells were analyzed for each condition in a single repeat (n = 3). Quantitative results represent the mean ± SD (One-way ANOVA: * *p* < 0.05; *** *p* < 0.001 versus control, ns: Not significant).

We next evaluated whether combining TP4 with EGFR-TKIs improves cytotoxicity in NSCLC cells. Cell lines with different EGFR status (EGFR-wild-type A549, EGFR-mutated NCI-H1975, and HCC827) were used to test the efficacy of combined treatments. In A549 cells, a combination of 10 μM Erlotinib (Erlo) or Gefitinib (Gef) with a range of TP4 concentrations (3.35–6.71 μM) showed enhanced cytotoxicity (Figure 2A). In NCI-H1975 cells, combinations of 10 μM of TKIs with TP4 (3.35–6.71 μM) showed enhanced cytotoxicity compared to TKI or TP4 treatments alone (0.11 ± 0.04, 0.06 ± 0.01, and 0.02 ± 0.01 fold in the Erlo + TP4 group; 0.14 ± 0.04, 0.06 ± 0.03, and 0.03 ± 0.01 fold in the Gef + TP4 group; and 0.32 ± 0.03, 0.07 ± 0.01, and 0.05 ± 0.02 fold in the TP4 alone group) (Figure 2B). In the HCC827 cells, a combination of 1 μM of TKIs with TP4 (3.35–6.71 μM) showed enhanced cytotoxicity compared to TKIs or TP4 treatment alone (0.32 ± 0.03, 0.15 ± 0.02 and 0.03 ± 0.01 fold in the Erlo + TP4 group; 0.26 ± 0.02, 0.14 ± 0.02 and 0.04 ± 0.01 fold in the Gef + TP4 group; and 0.65 ± 0.07, 0.47 ± 0.08 and 0.24 ± 0.07 fold in the TP4 alone group) (Figure 2C). These results showed that combination treatment markedly improved cytotoxicity in EGFR-mutated cells. However, combination treatment did not show better efficacy than TP4 alone in NSCLC cells with wild-type EGFR.

### 2.3. TP4 Induces Necrotic Death in NSCLC Cells

**Figure 3:**
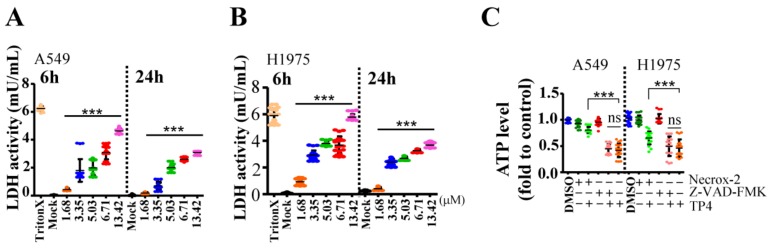
TP4 triggers NSCLC death by necrosis. (**A**,**B**) lactate dehydrogenase (LDH) release in A549 (**A**) and NCI-H1975 (**B**) cultures was determined 6 h or 24 h after treatment with varying doses of TP4 (1.68−13.42 μM). t-Octylphenoxypolyethoxyethanol (Triton-X) was used as a positive control. Each independent replicate was measured at least in triplicate (n = 3). Quantitative results represent the mean ± SD (One-way *ANOVA*:* *p* < 0.05; *** *p* < 0.001 versus control, ns: not significant). (**C**) Cell viability of A549 and H1975 cells were determined by the ATP assay 24 h after treatment with Dimethyl sulfoxide (DMSO), Necrox-2 (10 μM, Necrosis inhibitor), Z-VAD-FMK (50 μM, pan-caspase inhibitor), TP4 (6.71 μM), or combinations thereof. At least six wells were analyzed for each condition in a single repeat (n = 3). Quantitative results represent the mean ± SD (two-tailed Student’s t-test: *** *p* < 0.001 versus control, ns: Not significant).

**Figure 4:**
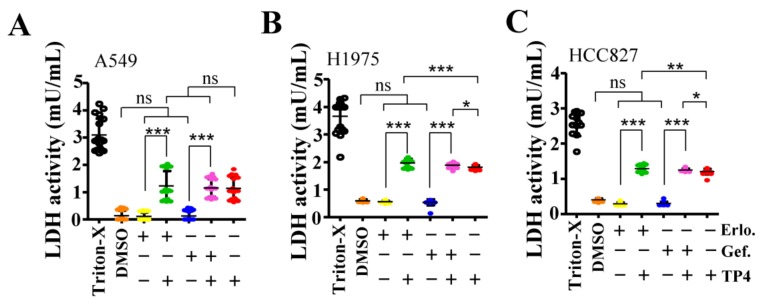
Combining TP4 with EGFR-TKIs enhances necrosis in EGFR-mutated NSCLC cells. (**A**–**C**) LDH release in A549 (**A**), NCI-H1975 (**B**), and HCC827 (**C**) cultures was determined 24 h after treatment with Triton-X, DMSO, EGFR-TKIs, TP4, or combinations thereof. Triton-X was used as a positive control. Each independent replicate was measured at least in triplicate (n = 3). Quantitative results represent the mean ± SD (two-tailed Student’s t-test: * *p* < 0.05; *** *p* < 0.001 versus control, ns: Not significant).

We next examined the cell death pathway triggered by TP4 in NSCLC cells. Treatment of TP4 for six and 24 h induced lactate dehydrogenase (LDH) release from NSCLC cells (Figure 3A,B), suggesting the occurrence of necrotic death. To evaluate whether apoptotic death may also be induced at early time-points after TP4 treatment (6.71 μM), we assayed caspase three activation and Lamin cleavage at 1.5 and three hours post drug treatment. The results showed no obvious changes in the levels of cleaved Lamin A/C, Lamin B1 or caspase three upon TP4 treatment (Supplementary Figure S1A,B). Moreover, treatment of cells with Necrox-2 (10 μM, Necrosis inhibitor) but not Z-VAD-FMK (50 μM, pan-caspase inhibitor) blocked TP4-induced cell death (Figure 3C). Together, these results indicate that TP4 robustly induces necrotic cell death in NSCLC cells. Furthermore, we asked whether combined TP4/TKI treatments also induce necrosis in NSCLC cells. The results showed that no significant difference in LDH production was observed in A549 cells after combined treatment (10 μM TKIs + 6.71 μM TP4); while a significant increase of LDH level was measured in H1975 or HCC827 cells with combined treatment (10 μM or 1 μM TKIs + 6.71 μM TP4) (Figure 4A–C). These results are consistent with the findings showing improved cellular toxicity of combination treatments in EGFR-mutated cells but not in EGFR-wild-type cells.

## 3. Discussion

In this work, we show that the antimicrobial peptide, TP4, shows excellent cytotoxicity toward NSCLC cells with different EGFR status, and combining TP4 with potent EGFR-TKIs enhanced cytotoxicity in EGFR-mutated cells. The ratio of surviving EGFR-mutated H1975 cells and HCC827 cells was decreased from 17.6−25.6% (10 μM TKIs) to 1.7%−13.6% (10 μM TKIs + 3.35−6.71 μM of TP4) and 47.1−50.7% (1 μM TKIs) to 3%−25.5% (1 μM TKIs + 3.35−6.71 μM of TP4) (Figure 1E,F), suggesting that these combinations may be considered as a potential therapeutic strategy for EGFR-mutated NSCLC. Similar responses were not observed in EGFR-wild-type A549 cells, where TP4 alone was sufficient to cause maximal cell death (Figure 1C). Furthermore, enhanced necrosis was observed in EGFR-mutated NSCLC cells after combination treatment (Figure 4B,C). While TKIs are known to induce apoptosis in cultured NSCLC cells, it has been reported that combined SU11274 (c-Met inhibitor) with Erlotinib resulted in tumor necrosis [22]. Dual effects induced by AMP in cancer cells have been reported [32]. High concentrations of AMPs may directly lyse membranes, while low concentrations of AMPs can induce controlled cell death (i.e., apoptosis, necroptosis, or others). Here, we found that TP4 mainly induced necrosis in NSCLC cells and not apoptosis (Figure 3A,B, and Supplementary Figure S1), since TP4-induced death was blocked by Necrorex-2 but not Z-VAD-FMK (Figure 3C). This result is similar to our earlier findings showing that TP4 induces necrotic death in triple-negative-breast-cancer (TNBC) cells [27]. Interestingly, combining EGFR-TKIs with TP4 enhanced cytotoxicity to EGFR-activated NSCLC cells (Figure 1E,F), suggesting that blockage of EGFR signaling contributes to TP4-mediated cytotoxicity. However, it remains unclear whether TP4 can modulate EGFR signaling in NSCLC cells. The neutrophil antimicrobial peptide LL37/hCAP-18 was shown to induce DNA breaks in A549 cells [33], with low concentrations (1 μg/mL) inducing EGFR transactivation to promote keratinocyte migration [34]. Thus, our study suggests a link between AMP action and EGFR activity.

The mechanism of TP4-mediated cytotoxicity may differ depending on the cancer cell type, transcriptome, or regulation of specific genes. For example, TP4 was found to induce FBJ murine osteosarcoma viral oncogene homolog B (FOSB) activation in all tested TNBC cells. Interestingly, in MDA-MB-231 and MCF7 cells, full-length FOSB is induced by TP4, while the alternatively spliced form (truncated) of FOSB (FOSΔB) is not; conversely, TP4 mainly induces FOSΔB in MB453 cells [27]. Moreover, either FOSB or FOSΔB overexpression triggered cell death in all tested breast cancer cells, and *FOSB* knockdown attenuated TP4-mediated cytotoxicity [27]. TP4 induction of *FOSB* (or *FOSΔB*) requires Ca^2+^ signaling and mitochondrial dysfunction to trigger necrotic death [27], suggesting that Ca^2+^-dependent FOSB signaling is involved in TP4-induced cytotoxicity. In addition, TP4 was shown to induce apoptosis in an osteosarcoma cell-line, MG63, through activation of extrinsic Fas/FasL- and intrinsic mitochondria-mediated pathways [35]. Pretreating these cells with Z-IETD-FMK (caspase-8 inhibitor) or Z-LEHD-FMK (caspase-9 inhibitor) significantly attenuated caspase-3 activation and prevented apoptosis [35]. These findings indicated that TP4 stimulates distinctive cytotoxic pathways among different cancer types. In NSCLC, we observed TP4-induced necrotic but not apoptotic death (Figure 3, Figure 4, and Figure S1), which is similar to what was previously found in TNBC cells. Whether FOSB signaling and Ca^2+^ homeostasis are also involved in TP4-mediated cytotoxicity in NSCLC cells remains to be further addressed.

The therapeutic efficacy of some AMPs, such as Xenopus skin-derived hymenochirin-1B and magainin, has been studied in lung cancer [36,37]. Hymenochirin-1B shows potent cytotoxicity in A549 cells with a lethal concentration (LC)_50_ of 2.5 μM. Meanwhile, magainin analogs (magainin A and G) were shown to exhibit antitumor activity in SCLC cell lines but were less-toxic to normal human fibroblast cell lines [38]. The average IC_50_ of MAG A and G in the SCLC cell lines were 8.64 and 8.82 µM, respectively, while the average IC_50_ of MAG A and G against normal human fibroblast cell lines were 21.1 and 29.2 µM, respectively. In addition, two sequence-modified magainin analogues (MSI-136 and MSI-238), which were designed to enhance the amphiphilic structure, showed more toxicity to A549 cells compared to magainin 2 [39]. The hymenochirin-1B and magainin analogues harbor seven positively charged amino acid residues, while TP4 contains ten positive charged residues. The positively charged residues of cationic AMPs electrostatically interact with anionic molecules on the cell membrane [40]. In addition, the number of positively charged residues has been correlated with amphipathicity and antimicrobial activity [40]. The in vitro cytotoxicity (IC_50_) of all _L_-form MSI-136 and all _D_-form MSI-238 to the A549 cells are 6 μg/mL (2.44 μM) and 10 μg/mL (4.06 μM), respectively. The all _L_-form of TP4 has an IC_50_ value about 1.922 μM (Table 2), indicating that TP4 is a more potent peptide drug than the magainin analogues and hymenochirin-1B. Intratumoral injection of TP4 in TNBC cell-xenotransplanted mice showed no adverse side-effects [27], however, strong hemolytic activity has been described for the molecule [29]. _D_-form amino acid-modified MSI-238 has been used to enhance proteolytic stability and to extend the survival of P388 tumor-bearing mice in vivo [40], providing a feasible approach for the development of modified TP4 isoforms for NSCLC therapy.

Overall, this study suggests that the tilapia-derived AMP, TP4, as a highly potent peptide drug that may be useful for NSCLC treatment. TP4 monotherapies can be used in NSCLC with wild-type EGFR, and combination therapies of TP4 with EGFR-TKIs may be applicable in EGFR-mutated NSCLC.

## 4. Material and Methods

### 4.1. Reagents and Peptide Sequence Analysis

TP4 (FIHHIIGGLFSAGKAIHRLIRRRRR) were synthesized and purified by GL Biochem Ltd. (Shanghai, China) as previously described [27]. Necrosis inhibitor, Necrox-2, and pan-Caspase inhibitor, Z-VAD(OMe)-FMK, were purchased from Santa Cruz Biotechnology Inc. (Dallas, TX, USA) *N*-(3-Chloro-4-fluoro-phenyl)-7-methoxy-6-(3-morpholin-4-ylpropoxy)quinazolin-4-amine (Gefitinib) and *N*-(3-ethynylphenyl)-6,7-bis(2-methoxyethoxy)-4-quinazolinamine hydrochloride (Erlotinib) were purchased from Sigma-Aldrich (St.Louis, MO, USA). Primary antibodies were purchased from the Cell Signaling (Danvers, MA, USA) (Lamin A/C, clone 4C11; Lamin B1, clone D4Q4Z; Caspase 3; cleaved Caspase 3) and EMD Millipore (GAPDH, clone 6C5) (Temecula, CA, USA). The Structural model and the helical projection of TP4 were generated, as previously described [41].

### 4.2. Cell Culture and Cell Viability Assay

Cell-lines (A549 (BCRC 60074), NCI-H661 (BCRC 60125), NCI-H209 (BCRC 60123), NCI-H1975 (ATCC CRL-5908), BEAS-2B (ATCC CRL-9609), and MRC-5 (BCRC 60023)) were purchased from the Bioresource Collection and Research Center (BCRC) (Taipei, Taiwan) and the American Type Culture Collection (ATCC) (Manassas, VA, USA) and cultured as suggested by BCRC and ATCC. For the cell viability assay, 4–5 × 10^3^ cells were seeded into the wells of a 96-well plate and cultured overnight. During the drug treatment assay, inhibitors were added 30 minutes prior to TP4, and cell viability was determined at indicated time-points. LDH release was quantified with a Cytotoxicity Detection Kit^PLUS^ (LDH) (Roche Applied Science, Basel, Switzerland), as previously described [27].

### 4.3. Western Blotting

For Western blots, sample preparations were performed, as previously described [28], and were electrophoresed and transferred onto polyvinylidene fluoride (PVDF) membrane. The membranes were incubated in blocking buffer (0.1 M PBS, 5% non-fat milk, 0.2% Tween-20) for 1 hour at room temperature and then incubated in the same solution with primary or secondary antibodies (GE Healthcare Life Science, Buckinghamshire, UK). Signals were detected by enhanced chemiluminescence (Immobilon Western Chemiluminescent HRP substrate, Merck Millipore, Billerica, MA, USA) on an imaging system (UVP, BioSpectrumTM 500 (Upland, CA, USA)).

### 4.4. Statistical Analysis

Cells were plated at least in quadruplicate for the multi-well based assay. Data were collected from repeated experiments (n ≥ 3) and were analyzed by Prism5 software (GraphPad Inc., (La Jolla, CA, USA)). Statistical significance was determined by two-tailed Student’s t-test or one-way analysis of variance (ANOVA) with Bonferroni post hoc test. Differences were considered statistically significant at *p* < 0.05.

## Figures and Tables

**Table 1:** IC_50_ values for TP4 in normal cells at various treatment times. Results are presented as mean ± SD.

Treatment	IC_50_ (μM)	95% Confidence Interval
**BEAS-2B**		
3 h	33.100 ± 1.032	31.11 to 35.22
6 h	32.040 ± 1.048	29.21 to 35.15
12 h	29.760 ± 1.074	25.88 to 34.22
24 h	26.500 ± 1.086	22.53 to 31.17
**MRC-5**		
3 h	46.440 ± 1.049	42.32 to 50.96
6 h	28.000 ± 1.068	24.65 to 31.80
12 h	18.220 ± 1.079	15.67 to 21.17
24 h	15.480 ± 1.063	13.72 to 17.46

**Table 2:** IC_50_ values for TP4 in NSCLC cells at various treatment times. Results are presented as mean ± SD.

Treatment	IC_50_ (μM)	95% Confidence Interval
**A549**		
3 h	27.620 ± 1.402	14.25 to 53.55
6 h	17.080 ± 1.236	11.27 to 25.89
12 h	4.089 ± 1.130	3.216 to 5.200
24 h	1.922 ± 1.112	1.560 to 2.367
**NCI-H661**		
3 h	14.170 ± 1.169	10.44 to 19.24
6 h	11.890 ± 1.102	9.824 to 14.38
12 h	5.276 ± 1.111	4.289 to 6.489
24 h	3.769 ± 1.113	3.054 to 4.652
**NCI-H1975**		
3 h	5.472 ± 1.112	4.445 to 6.737
6 h	3.262 ± 1.143	2.513 to 4.236
12 h	2.755 ± 1.202	1.920 to 3.954
24 h	1.241 ± 1.174	0.9065 to 1.698
**HCC827**		
3 h	18.520 ± 1.304	11.00 to 31.16
6 h	18.370 ± 1.153	13.89 to 24.29
12 h	16.070 ± 1.154	12.12 to 21.29
24 h	10.610 ± 1.146	8.118 to 13.86

## Supplementary Materials

Supplementary Figure S1, Table S1, and Table S2 are published online alongside the manuscript. Figure S1: TP4 does not induce caspase 3 activation and Lamin cleavage. (A, B) Total lysates from A549 (A) and NCI-H1975 (B) without (M) or with TP4 treatment (T) for 1.5 and 3 h were analyzed by Western blot using antibodies against GAPDH, caspase 3, and cleaved-caspase 3. Red dotted lines demarcate samples collected from three independent assays. Table S1: TP4 toxicity in normal BEAS-2B and MRC-5 cells was evaluated by the ATP assay. Statistical test results related to Figure 1A and B are shown. Eight wells were analyzed for each experiment (n = 24 per dose). Results represent the mean ± SD from independent experiments. Statistical comparisons between mock and each dose of TP4 were performed using one-way ANOVA analysis with Bonferroni post hoc test: ns, not significant; * *p* < 0.05; ** *p* < 0.01; *** *p* < 0.001. Table S2: TP4 toxicity in lung cancer cells was evaluated by the ATP assay. Statistical test results related to Figure 1C−F are shown. Multiple wells were analyzed for each experiment (*n* = 24 per dose in A549, H661, H1975 cells and *n* ≥ 17 per dose in HCC827 cells, respectively). Results represent the mean ± SD from independent experiments. Statistical comparisons between mock and each dose of TP4 were performed using one-way ANOVA analysis with Bonferroni post hoc test: ns, not significant; * *p* < 0.05; ** *p* < 0.01; *** *p* < 0.001.
