# Supplementary material for: Nile Tilapia Derived TP4 Shows Broad Cytotoxicity toward to Non-Small-Cell Lung Cancer Cells
Source: Mar Drugs. 2018 Dec 13;16(12):506. doi: 10.3390/md16120506 (PMC6316113; doi:10.3390/md16120506)
Supplement: Supplementary file 1 [file marinedrugs-16-00506-s001.pdf]

Supplementary Files

Nile tilapia derived TP4 shows broad cytotoxicity toward to non-small-cell lung cancer cells.

Chen-Hung Ting[1], Jyh-Yih Chen[1*]

1. Marine Research Station, Institute of Cellular and Organismic Biology, Academia Sinica, 23-10 Dahuen Road, Jiaushi, Ilan 262, Taiwan

* Corresponding author: Dr. Jyh-Yih Chen, Marine Research Station, Institute of Cellular and Organismic Biology, Academia Sinica, 23-10 Dahuen Road, Jiaushi, Ilan 262, Taiwan.

Tel: 886-920802111

Fax: 886-39871035

E-mail: zoocjy@gate.sinica.edu.tw

Supplementary Table

Supplementary Table S1. TP4 toxicity in normal BEAS-2B and MRC-5 cells was

Dose	0.84	1.68	3.35	5.03	6.71	13.42	20.12 (μM)
**BEAS-2B**							
3hr	1.07±0.09^{ns}	0.93±0.09^{ns}	0.72±0.07***	0.75±0.09***	0.69±0.05***	0.24±0.20***	0.05±0.01***
6hr	0.99±0.10^{ns}	1.06±0.07^{ns}	1.03±0.05^{ns}	0.96±0.13^{ns}	0.79±0.11***	0.11±0.03***	0.10±0.23***
12hr	1.20±0.11***	1.36±0.13***	1.46±0.30***	1.22±0.17***	1.16±0.23*	0.25±0.10***	0.03±0.01***
24hr	1.49±0.08***	1.58±0.12***	1.48±0.21***	1.38±0.23***	1.48±0.21***	0.16±0.07***	0.20±0.48***
**MRC-5**							
3hr	1.01±0.07^{ns}	0.99±0.06^{ns}	0.96±0.12^{ns}	0.84±0.07***	0.84±0.07***	0.65±0.12***	0.51±0.09***
6hr	1.02±0.06^{ns}	1.01±0.04^{ns}	0.85±0.09**	0.94±0.09^{ns}	0.94±0.09^{ns}	0.45±0.25***	0.31±0.20***
12hr	1.18±0.11***	1.17±0.13**	1.12±0.18^{ns}	1.07±0.15^{ns}	1.07±0.15^{ns}	0.24±0.22***	0.11±0.07***
24hr	1.08±0.11^{ns}	1.13±0.12***	1.08±0.11^{ns}	0.91±0.11*	0.91±0.11*	0.12±0.02***	0.05±0.02***

Supplementary Table S2.

Dose	0.84	1.68	3.35	5.03	6.71	13.42	20.12 (μM)
**A549**							
3hr	1.12±0.12^{ns}	0.86±0.22^{ns}	0.85±0.10 ^{ns}	0.74±0.30***	0.70±0.20***	0.06±0.04***	0.04±0.04***
6hr	1.00±0.07^{ns}	0.92±0.07^{ns}	0.77±0.17***	0.67±0.14***	0.51±0.22***	0.281±0.17***	0.02±0.02***
12hr	1.03±0.06^{ns}	1.04±0.09^{ns}	0.62±0.08***	0.41±0.14***	0.28±0.12*	0.15±0.15***	0.00±0.00***
24hr	1.15±0.08***	0.99±0.07^{ns}	0.50±0.09***	0.20±0.08***	0.17±0.07***	0.00±0.00***	0.00±0.00***
**H661**							
3hr	1.10±0.10^{ns}	1.01±0.11^{ns}	0.88±0.11^{ns}	0.59±0.07***	0.59±0.07***	0.14±0.20***	0.04±0.02***
6hr	1.01±0.05^{ns}	0.90±0.06***	0.78±0.03***	0.64±0.06***	0.45±0.13***	0.09±0.04***	0.02±0.00***
12hr	1.03±0.05^{ns}	1.00±0.06^{ns}	0.81±0.06***	0.32±0.07***	0.32±0.03***	0.03±0.01***	0.01±0.00***
24hr	0.88±0.05^{ns}	0.81±0.08***	0.59±0.05^{ns}	0.28±0.07*	0.28±0.07*	0.01±0.01***	0.00±0.00***
**H1975**							
3hr	1.02±0.13^{ns}	0.89±0.11***	0.68±0.08***	0.54±0.09***	0.24±0.04***	0.09±0.05***	0.00±0.00***
6hr	0.96±0.14^{ns}	0.89±0.09*	0.56±0.06***	0.39±0.09***	0.21±0.08***	0.13±0.17***	0.00±0.00***
12hr	0.99±0.24^{ns}	1.07±0.24^{ns}	0.61±0.12***	0.18±0.08***	0.07±0.03***	0.00±0.00***	0.00±0.00***
24hr	1.02±0.12^{ns}	0.95±0.09^{ns}	0.32±0.03^{ns}	0.07±0.01***	0.05±0.02***	0.00±0.00***	0.00±0.00***
**HCC827**							
3hr	1.09±0.21^{ns}	0.98±0.12^{ns}	0.80±0.12***	0.74±0.08***	0.62±0.09***	0.42±0.20***	0.13±0.07***
6hr	0.95±0.03***	0.86±0.10***	0.69±0.10***	0.58±0.05***	0.42±0.06***	0.16±0.08***	0.10±0.07***
12hr	0.90±0.05***	0.84±0.04***	0.77±0.06***	0.68±0.07***	0.44±0.13***	0.12±0.07***	0.02±0.01***

24hr	0.86±0.03***	0.76±0.05***	0.67±0.07***	0.51±0.11***	0.27±0.11***	0.09±0.05***	0.03±0.02***

Fig. S1

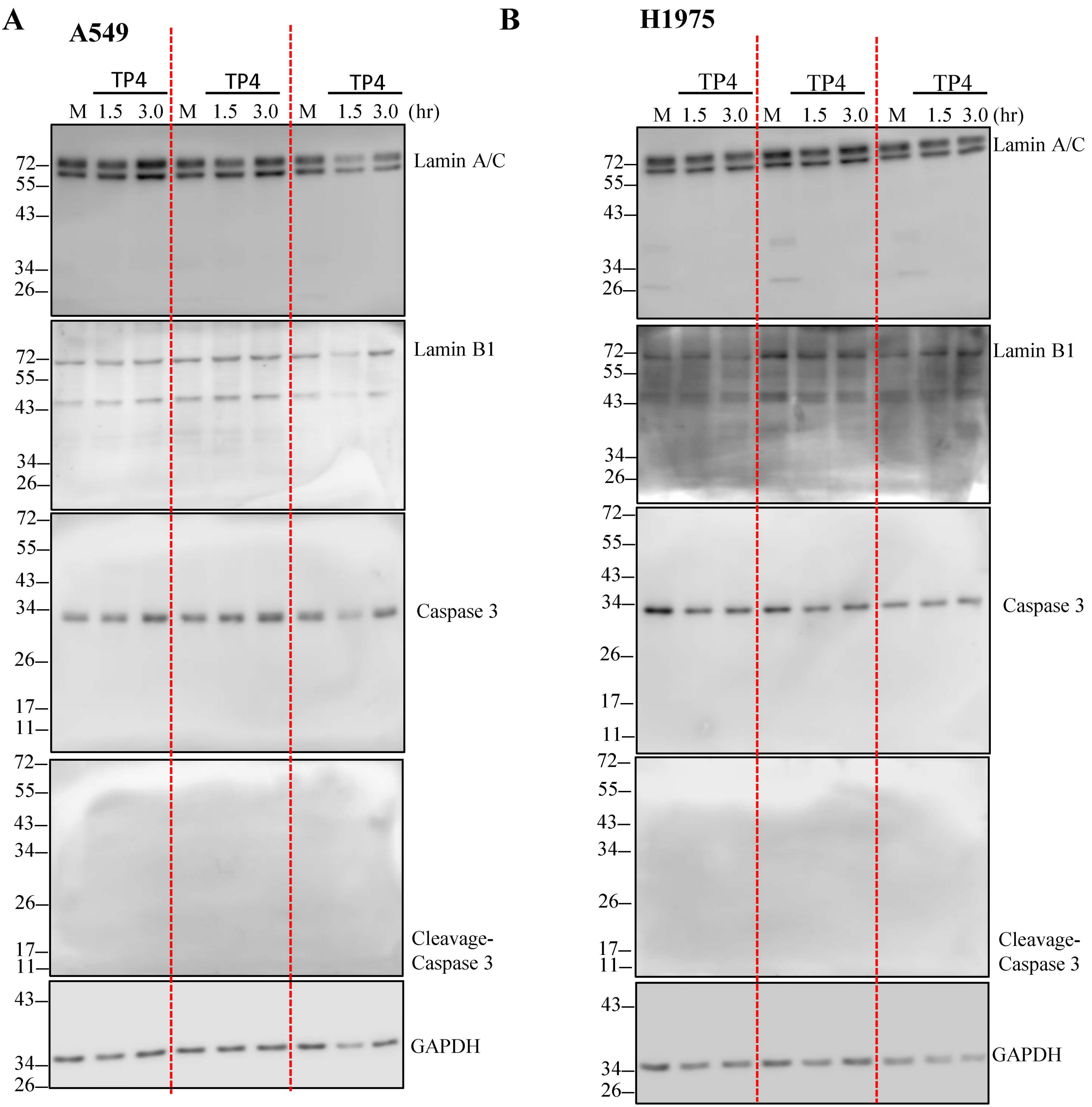
A
A549
TP4
M 1.5 3.0 M 1.5 3.0 M 1.5 3.0 (hr)
Lamin A/C
Lamin B1
Caspase 3
Cleavage-Caspase 3
GAPDH
72
55
43
34
26
17
11
B
H1975
